# Cost effectiveness of bilateral risk-reducing mastectomy and salpingo-oophorectomy

**DOI:** 10.1186/s40001-019-0391-8

**Published:** 2019-09-14

**Authors:** Michael G. Schrauder, Lisa Brunel-Geuder, Lothar Häberle, Marius Wunderle, Juliane Hoyer, Roland Csorba, André Reis, Rüdiger Schulz-Wendtland, Matthias W. Beckmann, Michael P. Lux

**Affiliations:** 1 Department of Gynecology and Obstetrics, Erlangen University Hospital, Comprehensive Cancer Center Erlangen-EMN, Friedrich-Alexander-Universität Erlangen-Nürnberg (FAU), Universitätsstraße 21–23, 91054 Erlangen, Germany; 2 Institute of Human Genetics, Comprehensive Cancer Center Erlangen-EMN, Friedrich-Alexander-Universität Erlangen-Nürnberg (FAU), Erlangen, Germany; 3 Institute of Radiology, Erlangen University Hospital, Comprehensive Cancer Center Erlangen-EMN, Friedrich-Alexander-Universität Erlangen-Nürnberg (FAU), Erlangen, Germany; 4 Department of Obstetrics and Gynecology, Hospital of Aschaffenburg-Alzenau, Aschaffenburg, Germany; 5 Faculty of Medicine, University of Debrecen, Debrecen, Hungary

**Keywords:** Breast cancer, Ovarian cancer, Genetic counseling, Cost effectiveness, *BRCA*

## Abstract

**Background:**

Growing demand for risk-reducing surgery in individuals with inherited susceptibility to cancer leads to the question whether these procedures are cost effective for the executing hospitals. This study compared the clinical costs for bilateral risk-reducing mastectomy (BRRM) with and without different types of reconstruction, risk-reducing salpingo-oophorectomy (RRSO), and their combinations with corresponding reimbursements in the statutory health-care system in Germany.

**Patients and methods:**

Real total costs of care for BRRM with and without reconstruction, RRSO, and their combinations were calculated as the sum of all personnel and technical costs. These costs calculated in a German University hospital were compared with the sum of all reimbursements in the German DRG-based health-care system.

**Results:**

While sole RRSO, BRRM without reconstruction, and BRRM with secondary DIEP (deep inferior epigastric perforator)—reconstruction still result in a small benefit, we even found shortfalls for the hospital with all other prophylactic operations under consideration. The calculated deficits were especially high for BRRM with implant-based breast reconstruction and for combined operations when the risk reduction is achieved with a minimum of separate operations.

**Conclusions:**

Risk-reducing surgery in *BRCA*-mutation carriers is frequently not cost-covering for the executing hospitals in the German health-care system. Thus, appropriate concepts are required to ensure a nationwide care.

## Background

The breast cancer 1 (BRCA1) gene, identified in 1994, and BRCA 2, identified in 1995, are the best known genetic risk factors for heritable breast cancer (BC) and ovarian cancer (OC) [1–5]. Heterozygous germline mutations in either *BRCA1* or *BRCA2* or other moderately to highly penetrant risk genes are responsible for about 5–10% of all cases of BC [6–9]. Nevertheless, the majority of patients currently diagnosed with BC or OC have a sporadic form of the disease. In the general population, the cumulative lifetime risk for BC and OC is 10–12% and about 1.5%, respectively. In contrast, *BRCA1* mutation carriers have a 60–65% risk for BC up to the age of 70 and a 40–70% risk for OC. For *BRCA2* mutation carriers, the corresponding percentages are 45–55% for BC and 11–16.5% for OC [7, 10]. In addition, mutation carriers have an increased risk of developing contralateral BC, relapses after initial BC treatment, pancreatic, and prostate cancer [2, 4, 9, 11–14].

The public recognition of a genetic predisposition to BC and OC has improved in recent years and an increasing number of women are seeking advice on this topic. In interdisciplinary Breast and Ovarian Cancer Genetics Clinics, different medical departments (gynecology, genetics, radiology, and psycho-oncology) collaborate in the effort to optimize evaluation and consultation for patients with positive family history or proven hereditary BC and OC. Guidelines published by several associations—including the National Comprehensive Cancer Network (NCCN), the American Society of Clinical Oncology (ASCO), and the German Consortium for Hereditary Breast and Ovarian Cancer (GC-HBOC)—have defined groups of unaffected individuals in whom genetic testing is appropriate and recommended [2–5, 15].

Generally speaking, risk-reducing strategies comprise structured intensified surveillance, chemoprevention, lifestyle factors, and risk-reducing surgeries. The risk-reducing surgical options in high-risk women include risk-reducing (bilateral) salpingo-oophorectomy (RRSO), bilateral risk-reducing mastectomy (BRRM), and contralateral risk-reducing mastectomy (CRRM) in those women already diagnosed with BC. The incidence of BC in healthy *BRCA*-mutation carriers can be reduced by at least 90% through BRRM. RRSO in premenopausal women reduces not only the incidence and mortality of ovarian and fallopian tube cancer by 90–96%, but also most studies have also shown a risk reduction for BC by at least 50% [16–20]. For patients expressing the wish to get a breast reconstruction, this surgical procedure can be performed “immediate” in the same operation together with BRRM or “delayed” in a second operation. For our economic analyses, we considered the most frequently used techniques for breast reconstruction in our hospital: immediate and delayed implant-based breast reconstruction as well as immediate and delayed DIEP (deep inferior epigastric perforator)-based breast reconstruction.

The objectives of risk-reducing surgeries (RRS) are to prevent diseases and thereby preserve health and gain life years in good health. It is indisputable that these aspects of RRS are most important, but with increasing cost pressure in hospitals, budget caps, and health-care saving plans the question remains, whether these procedures are cost-covering for the service-providing hospitals in Germany [21]. In a previously published cost-effectiveness analysis, we were able to show that RRS (BRRM and RRSO) in *BRCA*-mutation carriers is cost effective from the long-term statutory health insurance perspective in the German health-care system. Comparing health-care costs for RRS with potentially avoidable cancer treatment costs, we were able to demonstrate that all risk-reducing surgical procedures were cost effective. A potential cost reduction for the healthcare system of € 136,295 was calculated if BRRM had been performed and € 791,653 if RRSO had been performed before the development of cancer in only 50% of a defined group of 70 mutation carriers seen in our center between 2009 and 2013. Moreover, in patients with combined RRSO and BRRM (without breast reconstruction), one further life year for a 40-year-old BRCA-mutation carrier would cost € 2183 based on our mono-centric calculations. Considering the costs per life year gained, we calculated that combined BRRM and RRSO is the most cost-effective approach, followed by BRRM alone [22, 23].

The fact that national long-term health-care costs can be reduced by risk-reducing surgeries after genetic testing in BRCA-mutation carriers does not allow any conclusion about whether RRS are cost-covering for the hospitals in Germany offering such services. Therefore, the purpose of this study was to examine the cost effectiveness of risk-reducing surgeries in individuals with a proven *BRCA* mutation in the context of a University Hospital with certified Breast Cancer Center in Germany.

## Patients and methods

### Study design and genetic testing

The study was conducted in the interdisciplinary Breast and Ovarian Cancer Genetics Clinic at the University Breast Center for Franconia in Erlangen, Germany. The study group (*n* = 370) comprised all individuals seen at our Genetics Clinic between 2009 and 2013. Data for all these individuals were collected retrospectively from their medical records. Individuals fulfilling the inclusion criteria for genetic testing were offered genetic counseling and germline mutation testing. If a germline BRCA pathogenic mutation was detected, we offered RRS and performed a comprehensive informed consent discussion.

### Defining hospital costs for RRS

All costs in this analysis are described in euros (€) (2012 value). Costs were calculated from the perspective of a German University Hospital with certified Breast Cancer Center offering RRS. All hospital costs arising for the risk-reducing surgical procedures under consideration (BRRM, RRSO with and without reconstruction) were calculated per person. We included all healthcare costs starting from the hospital admission for RRS to the hospital discharge after the last (e.g., reconstructive) surgery. In this process, total costs of care for a certain risk-reducing surgical procedure were defined as the sum of personnel, material, and technical costs in the hospital. Additional costs for potential postoperative complications were not included. Costs of prophylactic risk-reducing surgery were collected based on cost-unit accounting. The entire treatment of typical index patients who had undergone BRRM, RRSO, or simultaneous BRRM and RRSO operations with and without breast reconstruction was surveyed in consideration of all parts of the preoperative, operative, and postoperative care. In all separate areas of the hospital personnel cost, material costs, incidental expenses, and costs for infrastructure were separately calculated. All costs were listed and averaged. Thereby, the following areas of patient care were considered: ward, laboratory examination, cardiology, anesthesia, operation theatre, and intensive care unit.

### Personnel costs

Personnel were grouped in certain remuneration categories based on data provided by the Department of Human Resources of the Erlangen University Hospital. The expenditure of time for a patient with risk-reducing surgery was measured exemplarily in representatives of every category and for every type of risk-reducing operation under consideration. This was repeated three times and the results were averaged. The incurring personnel costs per operation were calculated as the sum of the personnel cost of all involved professional groups in the aforementioned hospital areas. The costs for each group were calculated by multiplying the average time consumption in minutes with the revenue per minute in this occupational group. Detailed lists and information regarding remuneration was provided by the Department of Human Resources of the Erlangen University Hospital Management.

### Material costs

The average material cost per patient for each risk-reducing procedure under consideration was calculated including medication, implants in case of implant-based breast reconstruction, and all further material costs (miscellaneous). The entire treatment of typical index patients who had undergone risk-reducing operations was surveyed and the average material costs in the three different categories were calculated.

### Revenue of the hospital for RRS

The expenditures of the hospital were opposed to the reimbursements for the RRS in the German hospital pricing system, based on diagnosis-related groups (DRG) and the operations and procedures keys (OPS) of the year 2012. The real accrued expenses for our hospital services associated with the considered surgical procedures were subtracted from revenues based on reimbursements by the DRG-based hospital pricing system considering the diagnoses (DRG) and the performed surgical procedures (OPS codes). The total DRG-based revenues were compartmentalized based on the InEK (“Institut für das Entgeltsystem im Krankenhaus” institute for the hospital payment system in Germany) calculations for the direct comparison of real partial costs in the hospital with revenues for personnel, material, and hospital infrastructure.

### Cost-unit accounting

Based on a cost carrier piece bill (“Kostenträgerstückrechnung”), all costs occurring in the hospital in connection with the treatment of one patient (one DRG) were calculated and compared with reimbursements. The InEK provides an allocation of DRG-related hospital reimbursements to the cost centers in the hospital (personnel, material, infrastructure, and technical costs). These separated proportions of a DRG-related reimbursement for the hospital were compared with the corresponding real costs in our hospital for these services. The comparisons were done for all risk-reducing surgical procedures under consideration: BRRM and the possible combinations with immediate and delayed breast reconstruction as well as RRSO.

## Results

### Patient characteristics

Between 2009 and 2013, 370 individuals were seen at the interdisciplinary Breast and Ovarian Cancer Genetics Clinic at the University Breast Center for Franconia. The majority of individuals were female (*n* = 362, 97.8%). The average age at time of presentation was 42 years with an age range of 18–85 years.

A proportion of individuals fulfilling diagnostic criteria opted out of genetic testing, leading to 242 individuals for genetic testing of *BRCA1* and *BRCA2* genes. Seven of the eight men were tested, and 235 of the 362 women. Genetic testing identified 70 (29%) known BRCA mutations. Of these 242 individuals, 44 (18%) were *BRCA1* and 26 (11%) *BRCA2* mutation carriers. Moreover, in 23 individuals (10%), variants of unknown significance were identified, seven in *BRCA1* and 16 in *BRCA2*. In total, 61% of individuals were tested completely negative for either *BRCA* gene.

#### Cost-unit accounting for RRS

##### Personnel costs

The personnel costs as a summary of medical services associated with different risk-reducing surgical procedures compared with revenues (cost-unit accounting) are summarized in Table 1. The costs are presented for the different professional groups (physicians, nursing, medical–technical, and total personnel costs). We were able to show that in our university hospital, the real personnel costs are covered for BRRM (€ 1359) and RRSO (€ 376) and well covered for BRRM with delayed DIEP-based breast reconstruction (€ 3520), but are not covered for BRRM with immediate implant-based breast reconstruction (€ − 1987), BRRM and delayed implant-based breast reconstruction (€ − 768), and BRRM with immediate DIEP-based breast reconstruction (€ − 722). In summary, for three out of the six risk-reducing surgical procedures under consideration, the personnel costs are not covered by the DRG-based reimbursements.

**Table 1:** Personnel costs compared with revenue (cost-unit accounting) in different professional groups for risk-reducing surgical procedures (negative values are represented in italics)

Operations	Personnel costs (cost-unit accounting) for hospital services			
	Medical	Nursing	Med.–Tech.	Total costs
BRRM	€ 670	€ 180	€ 509	€ 1359
RRSO	€ 307	€ 25	€ 45	€ 376
BRRM with immediate implant-based breast reconstruction	*€* *−* *459*	*€* *−* *1521*	*€ −* *6*	*€ −* *1987*
BRRM and delayed implant-based breast reconstruction	*€* *−* *136*	*€* *−* *362*	*€* *−* *270*	*€ −* *768*
BRRM with immediate DIEP-based breast reconstruction	*€* *−* *571*	€ 608	*€* *−* *759*	*€ −* *722*
BRRM with delayed DIEP-based breast reconstruction	€ 1802	€ 501	€ 1216	€ 3520

*BRRM* bilateral risk-reducing mastectomy, *RRSO* risk-reducing (bilateral) salpingo-oophorectomy, *DIEP* deep inferior epigastric perforator flap

##### Material costs

A similar pattern was seen for material costs, Table 2. While the refunded material costs for BRRM, RRSO, and BRRM with immediate implant-based breast reconstruction and BRRM with delayed DIEP-based breast reconstruction were slightly higher than the real material costs in our hospital (€ 753, € 90, € 619, and € 347), the real material costs for BRRM with delayed implant-based breast reconstruction (€ − 2786) and BRRM with immediate DIEP-based breast reconstruction (€ − 287) were not completely reimbursed by the DRG-based hospital pricing system.

**Table 2:** Material costs compared with revenue (cost-unit accounting) for risk-reducing surgical procedures (negative values are represented in italics)

Operations	Material costs (cost-unit accounting)			
	Medicines	Implants	Miscellaneous	Total costs
BRRM	€ 78	€ 136	€ 540	€ 753
RRSO	€ 22	€ 1	€ 67	€ 90
BRRM with immediate implant-based breast reconstruction	*€* *−* *39*	€ 579	€ 79	€ 619
BRRM and delayed implant-based breast reconstruction	€ 7	*€* *−* *2950*	€ 157	*€ −* *2786*
BRRM with immediate DIEP-based breast reconstruction	*€* *−* *1*	*€* *−* *458*	€ 172	*€ −* *287*
BRRM with delayed DIEP-based breast reconstruction	€ 80	*€* *−* *288*	€ 556	€ 347

*BRRM* bilateral risk-reducing mastectomy, *RRSO* risk-reducing (bilateral) salpingo-oophorectomy, *DIEP* deep inferior epigastric perforator flap

##### Costs for hospital infrastructure

The calculation of hospital infrastructure costs is divided in medical and non-medical costs as well as their summary (total costs for hospital infrastructure) (Table 3). That covers all costs for hospital infrastructure from heating and electricity to the wear of equipment. Again, the real costs for hospital infrastructure in our university hospital associated with BRRM with immediate implant-based breast reconstruction and for BRRM with delayed implant-based breast reconstruction were clearly higher than the partial costs calculated for hospital infrastructure in the InEK calculations systems. The differences were as high as € − 2137 for BRRM with immediate implant-based breast reconstruction and € − 917 for BRRM with delayed implant-based breast reconstruction, showing that even in a well-organized university hospital with a high number of cases and a high degree of capacity utilization the amount of money refunded for hospital infrastructure is lower than the real costs for some risk-reducing operations. For the other surgical procedures under consideration, our hospital costs for infrastructure were slightly lower than the refunds resulting in a surplus ranging from € 148 (RRSO) to € 475 (BRRM).

**Table 3:** Cost-unit accounting of medical and non-medical hospital infrastructure for risk-reducing surgical procedures compared with corresponding revenues (negative values are represented in italics)

Operations	Costs for hospital infrastructure (cost-unit accounting)		
	Medical	Non-medical	Total costs
BRRM	€ 155	€ 320	€ 475
RRSO	€ 63	€ 85	€ 148
BRRM with immediate implant-based breast reconstruction	*€* *−* *320*	*€* *−* *1817*	*€* *−* *2137*
BRRM and delayed implant-based breast reconstruction	*€* *−* *346*	*€* *−* *571*	*€ −* *917*
BRRM with immediate DIEP-based breast reconstruction	*€* *−* *233*	€ 390	€ 157
BRRM with delayed DIEP-based breast reconstruction	€ 183	€ 985	€ 148

*BRRM* bilateral risk-reducing mastectomy, *RRSO* risk-reducing (bilateral) salpingo-oophorectomy, *DIEP* deep inferior epigastric perforator flap

##### Structure of total hospital costs and comparison with revenues

Table 4 presents the summary of total costs for personnel, material, and infrastructure calculated by cost-unit accounting (calculated as differences between revenues and corresponding hospital costs) for the considered risk-reducing surgical procedures and their potential combination with reconstructive procedures. Half of the surgical procedures result in a deficit for the hospital providing the service. The highest shortfalls for the hospital are achieved with BRRM and delayed implant-based breast reconstruction (€ − 4471) followed by BRRM with immediate implant-based breast reconstruction (€ − 3504), and BRRM with immediate DIEP-based breast reconstruction (€ − 852).

**Table 4:** Summary of total costs (cost-unit accounting) for risk-reducing surgical procedures compared with total revenues (negative values are represented in italics)

Operations	Total hospital costs (cost-unit accounting)			
	Personnel	Material	Infrastructure	Total costs
BRRM	€ 1359	€ 753	€ 475	€ 2588
RRSO	€ 376	€ 90	€ 148	€ 614
BRRM with immediate implant-based breast reconstruction	*€* *−* *1987*	€ 619	*€* *−* *2137*	*€ −* *3504*
BRRM and delayed implant-based breast reconstruction	*€* *768*	*€* *−* *2786*	*€* *−* *917*	*€ −* *4471*
BRRM with immediate DIEP-based breast reconstruction	*€* *−* *722*	*€* *−* *287*	€ 157	*€ −* *852*
BRRM with delayed DIEP-based breast reconstruction	€ 3520	€ 347	€ 1168	€ 5035

*BRRM* bilateral risk-reducing mastectomy, *RRSO* risk-reducing (bilateral) salpingo-oophorectomy, *DIEP* deep inferior epigastric perforator flap

The direct comparison of total hospital costs and revenues (Table 5) makes it obvious that the revenues in the German DRG-based hospital pricing system are comparable low except for BRRM with delayed DIEP-based breast reconstruction. This fact results in shortfalls for the performing hospital in case of more elaborate and expensive procedures such as BBRM with delayed implant-based breast reconstruction. For this risk-reducing operation, the total expenses of € 13206 accrue in our hospital and are accompanied by total revenues of only € 8735 in the German DRG system (Table 5).

**Table 5:** Direct comparison of total hospital costs and revenues from the DRG reimbursement system for risk-reducing surgical procedures in a University hospital in the Germany (negative values are represented in italics)

	Costs	Revenues	Differences
BRRM	€ 4031	€ 6619	€ 2588
RRSO	€ 2161	€ 2775	€ 614
BRRM with immediate implant-based breast reconstruction	€ 10409	€ 6905	*€ −* *3504*
BRRM and delayed implant-based breast reconstruction	€ 13206	€ 8735	*€* *−* *4471*
BRRM with immediate DIEP-based breast reconstruction	€ 14470	€ 13618	*€ −* *852*
BRRM with delayed DIEP-based breast reconstruction	€ 15199	€ 20234	€ 5035

*BRRM* bilateral risk-reducing mastectomy, *RRSO* risk-reducing (bilateral) salpingo-oophorectomy, *DIEP* deep inferior epigastric perforator flap

The financial difficulties for German hospitals performing BRRM and RRSO in one simultaneous operation are outlined in Table 6. This table compares total hospital costs and revenues for BBRM combined with RRSO with and without different types of breast reconstruction. It is first evident that the patients’ benefits of getting all necessary and medically indicated procedures done in one operation which avoid additional narcoses and start earlier with the recovery phase are standing opposite to the shortfalls for the hospital. These hospital deficits are increasing with the complexity and the number of simultaneously performed procedures and is worst for BBRM with simultaneous RRSO followed by delayed implant-based breast reconstruction (€ − 6632) (Table 6).

**Table 6:** Comparison of total hospital costs and revenues from the DRG reimbursement system for different combinations of risk-reducing surgical procedures including simultaneous RRSO (negative values are represented in italics)

	Costs	Revenues	Differences
Simultaneous BRRM + RRSO	€ 6190	€ 6619	€ 429
BRRM + RRSO with immediate implant-based breast reconstruction	€ 12569	€ 6905	*€ −* *5664*
BRRM + RRSO and delayed implant-based breast reconstruction	€ 15367	€ 8735	*€* *−* *6632*
BRRM + RRSO with immediate DIEP-based breast reconstruction	€ 16630	€ 13618	*€ −* *3012*
BRRM + RRSO with delayed DIEP-based breast reconstruction	€ 17360	€ 20234	€ 2874

*BRRM* bilateral risk-reducing mastectomy, *RRSO* risk-reducing (bilateral) salpingo-oophorectomy, *DIEP* deep inferior epigastric perforator flap

## Discussion

The present study provides an economic analysis of risk-reducing surgery carried out at a university hospital in Germany. We revealed that operations in *BRCA-*mutation carriers are mostly not cost-covering for the executing hospital; especially if the risk reduction is achieved with a minimum of separate operations and if implant-based breast reconstruction is performed. While sole RRSO, BRRM without reconstruction, and BRRM with secondary DIEP reconstruction still result in a benefit of € 614; € 2590, and € 5035 per patient for the hospital, we found shortfalls for the hospital with all other prophylactic operations under consideration. We calculated a deficit of € − 3504 and € − 852 for BRRM with simultaneous implant-based and simultaneous DIEP-based reconstruction, as well as € − 4471 for BRRM with secondary implant-based reconstruction. The combination of these operations with simultaneous RRSO even increased the deficit for the performing hospital (Tables 5, 6).

With regard to genetic counseling and testing, different studies found cost effectiveness of counseling and testing based on clinical criteria and family history [24]. Recent publications showed that even a population-based panel testing for high- and moderate-penetrance OC and BC gene mutations in the US and UK population is cost effective (ICER = $ 54,769.78/QALY and £ 21,599.96/QALY) preventing around 1.9% of BC and 4.88% (US) and 3.2% (UK) of OC cases. The authors were even able to show that population-based BRCA1/BRCA2/RAD51C/RAD51D/BRIP1/PALPB2 panel testing is more cost effective than any clinical or family history-based testing of the same genes [25]. In a previously published cost-effectiveness analysis, we were able to show that BRRM and RRSO in BRCA-mutation carriers are cost-effective procedures in the German health-care system [22].

For patients with unilateral non-hereditary BC who underwent contralateral prophylactic mastectomy hospital, cost calculations in the US Medicare reimbursement system have previously been published. A mono-centric study reported that the increase in short-term healthcare costs for women receiving immediate contralateral prophylactic mastectomy (CPM) was $ 6528 and $ 16,744 for delayed CPM. The mean total reimbursements for unilateral BC treatment including immediate CPM (from the date of primary surgery to 24 months) were calculated based on US Medicare reimbursement to be $ 65,796 [26].

In contrast, the costs arise for the health-care system in Germany in the form of reimbursements for single BRRM without reconstruction amount to only € 6619 in the statutory DRG system in Germany. The revenues for BRRM with immediate implant-based breast reconstruction in Germany are only € 6905 and thereby only € 286 higher than for the already very low BRRM procedure alone. It is obvious that the additional effort for breast reconstruction can never be completely compensated by this amount.

These hospital costs contrast with the huge treatment costs for potential subsequent cases of BC and/or OC without risk-reducing surgeries. Assuming a situation in which 70 individuals with proven *BRCA* mutations were cancer-free and accepted BRRM and RRSO, it would be statistically possible to prevent the occurrence of at least 38 cases of BC and 19 cases of OC by carrying out risk-reducing surgeries, as shown in our previous publication. This corresponds to cancer treatment costs in Germany of nearly € 2.0 million that could be saved by carrying out BRRM and RRSO in 70 individuals [22, 27]. On the other hand, if all 70 individuals with proven *BRCA* mutations would have had BRRM with immediate implant-based breast reconstruction, the economic loss for our hospital due to the poor reimbursement of these operations would have reached € − 245280.

The combination of different risk-reducing surgical procedures offers the potential advantage of a single operation with a single postoperative recovery. With regard to secondary costs associated with loss of working hours and salary, a single operation would also allow the patient to return to work faster and save money in a socio-economic manner. Due to sterility—for example, in patients with implant-based breast reconstruction—or other medical aspects, not all procedures should be performed simultaneously, but often, simultaneous procedures are possible without reservation.

Cost analysis of high-risk patients undergoing simultaneous RRSO and BRRM with free flap breast reconstruction at the Hospital of the University of Pennsylvania verified that average total hospital costs were significantly higher in the group of patients receiving simultaneous surgery compared to patients without a combined gynecologic procedure [28]. In a multivariate regression with total cost as the dependent variable, the factor “simultaneous gynecologic procedure” predicted increased total costs [28].

Different studies reported higher costs for immediate breast reconstruction in different clinical settings, but the utility reported by patients was greater with immediate reconstruction [29–32]. The objective of immediate post-mastectomy breast reconstruction is to minimize deformity and optimize quality of life, especially in patients who do not want to undergo the experience of losing their breasts, even for a certain period of time before delayed reconstruction. The incomprehensible hospital pricing system in Germany—resulting in a hospital deficit for certain risk-reducing surgical procedures—makes the counseling situation for *BRCA*-mutation carriers in Germany unnecessarily complex. It creates crucial conflicts for the consulting physicians between financial matters and patient preferences. In this perspective, the hospital reimbursement system in Germany with considerable higher shortfalls for the hospital in case of simultaneously performed surgical procedures (Table 6) seems to be inappropriate and ethically questionable.

However, our findings need to be interpreted in the light of several limitations. First, the study was conducted a few years ago at a single site, so that hospital-level variations and recent increases in reimbursements are not taken into account. Second, the calculations did not include the secondary and tertiary costs associated with potential complications and revision surgery. Revisions in implant-based breast reconstructions are of particular relevance in relation to health-care costs. The overall revision rate in implant-based breast reconstruction is thought to be about 20% and it is, therefore, a relevant cost factor [33]. However, costs for revisions are difficult to estimate, accumulate over a very long period of time, and were, therefore, not included in the present cost calculations. Especially, notable is that all expenses for complications or revisions dramatically deteriorate the already poor cost effectiveness of most risk-reducing surgical procedures.

It could be argued that shortcomings in the infrastructure and organization of the considered hospital are responsible for higher hospital costs compared to other national and international hospitals. In comparison, published estimates of prophylactic and therapeutic surgical costs based on actuarial data from the University Hospital of Cologne describe costs for prophylactic mastectomy € 8317 and prophylactic oophorectomy € 2854 based on data from 2012 to 2014 [34]. There are further studies calculating these costs in US and UK hospitals. For example, the costs for unilateral mastectomy or BRRM, defined as sole institutional costs for operating room and facility costs (without physician fees), were calculated as high as $ 7, 718 (unilateral) and $ 11,992 (bilateral mastectomy) in an US breast center in 2008 [35]. Published cancer care costs in American hospitals were integrated in the publication of Grann et al. describing total costs (direct and indirect) for prophylactic mastectomy $ 10,591 and prophylactic salpingo-oophorectomy $ 6373 [36, 37]. Another study reported total hospital costs without professional service fees or charges for BRRM with immediate DIEP-based breast reconstruction of $ 20,516 and in combination with RRSO and hysterectomy of $ 23,862 for patients treated between 2005 and 2012 [28]. The overall mean cost for BRRM with immediate implant-based breast reconstruction in an UK hospital in Winchester was £ 14,797 per patient treated between 1991 and 2011 [38]. All these studies reported higher hospital costs for the risk-reducing surgical procedures under consideration compared to the calculated costs in our University hospital within the German healthcare system.

## Conclusion

Despite several limitations, this is the first study to calculate health-care hospital costs arising for risk-reducing surgeries in *BRCA-*mutation carriers at a single university hospital in Germany. It demonstrates serious deficits in the German reimbursement system for risk-reducing surgeries and provides important data for further research and health-economic assessments in this area of the German health-care system.

## Data Availability

All data generated or analyzed during this study are included in this published article.

## Abbreviations

ASCO: American Society of Clinical Oncology
BC: breast cancer
*BRCA1*: Breast cancer 1 (gene)
DRG: diagnosis-related group(s)
GC-HBOC: German Consortium for Hereditary Breast and Ovarian Cancer
NCCN: National Comprehensive Cancer Network
NICE: National Institute for Health and Care Excellence
OC: ovarian cancer
BRRM: bilateral risk-reducing mastectomy
RRS: risk-reducing surgery
RRSO: risk-reducing (bilateral) salpingo-oophorectomy
CRRM: contralateral risk-reducing mastectomy
QALY: quality-adjusted life years
